# BioMutation: a portable graphical user interface for mutagenesis and feature analysis in proteins, nucleic acids, and their complexes

**DOI:** 10.3389/fgene.2026.1769896

**Published:** 2026-03-17

**Authors:** Tushar Gupta, Pradeep Pant

**Affiliations:** Department of Biotechnology, Bennett University, Greater Noida, India

**Keywords:** computational modeling, GUI (graphical user interface), mutagenesis, nucleic acid mutation, protein mutation

## Abstract

Protein and nucleic acid mutational studies are central to understanding biomolecular structure, function, and interactions, yet existing computational tools often lack user-friendly interfaces for high-throughput and systematic mutagenesis. To address this limitation, we present BioMutation, a graphical user interface (GUI) that enables automated, batch-wise introduction of substitution-based mutations in proteins, nucleic acids, and their complexes facilitated via UCSF ChimeraX functionalities. BioMutation supports user-defined and class-wise amino acid substitutions in proteins, as well as user-specified and combinatorial substitutions in DNA and RNA. The tool automatically generates libraries of mutated structures in standard formats (PDB, MOL2, and mmCIF) suitable for downstream computational studies, including molecular docking and molecular dynamics simulations. In addition, BioMutation includes a Google Colab–based Structure Analyzer module for comparative assessment of physicochemical features before and after mutation. By integrating automation, flexibility, and accessibility within a single platform, BioMutation facilitates efficient *in silico* mutagenesis for applications in structural biology, biomolecular engineering, and drug discovery. The GUI is freely available at https://github.com/Computational-biolab/BioMutation.

## Graphical Abstract

**Figure:**
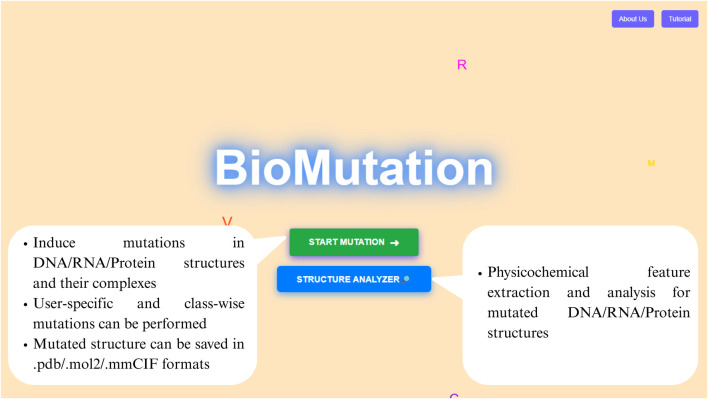

## Introduction

The structural integrity and functionality of biomolecules are intrinsically governed by the sequence and arrangement of their constituent building blocks (Bao, 2002). Any alteration in these fundamental units can significantly impact the overall structure, behaviour, and biological roles of the biomolecule (Arnittali et al., 2019). In the case of nucleic acids (DNA and RNA), the fundamental monomeric unit is the nucleotide, comprising a five-carbon sugar, a phosphate group, and a variable nitrogenous base (Pauling and Corey, 1953; Sim et al., 2012; Zamenhof, 1967). Mutations in the nitrogenous base region can result in considerable changes in the structure and function of the nucleic acid molecule (Zamenhof, 1967). Similarly, proteins are composed of amino acids, which serve as both structural and functional units (Efimov, 1993). Each amino acid consists of a central carbon atom bonded to a hydrogen atom, an amino group, a carboxyl group, and a unique side chain or R-group (Efimov, 1993; Reva et al., 2011). Variations in these side chains give rise to a diverse range of amino acids, which in turn contribute to the formation of functionally distinct proteins (Feyfant et al., 2007). Alterations in the amino acid sequence or in the nucleotide sequence of DNA or RNA can lead to changes in biomolecular orientation and function. These changes, collectively referred to as mutations, can occur naturally or be introduced synthetically (Auerbach, 2013). Such mutations may result in functional loss or gain or produce effects that have wide-ranging implications, from beneficial applications in research and industry to severe pathological outcomes, including genetic disorders and diseases (Chakarov et al., 2014; Soskine and Tawfik, 2010; Studer et al., 2013; Tokuriki and Tawfik, 2009).

To explore and understand the implications of mutations, it is crucial to develop computational tools that enable researchers to introduce and study mutations *in silico*. The process of *in silico* mutagenesis allows researchers to systematically explore multiple modified residues and may help them evaluate how specific modifications may influence the structural and functional properties of biomolecules (Carter, 1986; Auerbach, 1949). Mutagenesis has demonstrated substantial value across diverse scientific domains. In agriculture, for example, it is employed to enhance crop resistance to pests, diseases, and environmental stressors (Botstein and Shortle, 1985; Oladosu et al., 2016), as well as to improve yield and nutritional quality (Animasaun and Oguntoye, 2024). In animal husbandry, mutagenesis techniques contribute to genetic improvement of livestock, development of disease-resistant breeds, and enhancement of product quality (Jiang and Shen, 2019). In the realm of biotechnology and medical research, mutagenesis aids in enzyme engineering, drug discovery, target validation, and the advancement of personalized medicine (Mahdieh and Rabbani, 2013; Rowlands, 1984; Ahloowalia et al., 2004; Bikker et al., 2009). It also supports the study of protein evolution and phenotypic diversity, as well as the engineering of enzymes for therapeutic use (Friedman, 2022). Additionally, mutations play a role in redesigning of metabolic pathways and development of biosensors (Parsons et al., 2005; Berdan et al., 2021). Despite these applications, experimental mutagenesis studies are often constrained by several limitations (Smith, 1992; Tang et al., 2017). Generating multiple variants through site-directed or random mutagenesis is time-consuming, resource-intensive, and often technically demanding (Koh et al., 1997; Ling and Robinson, 1997; Auerbach, 1951). The characterization of these mutants requires laborious expression, purification, and analysis steps, each of which may be prone to variability and failure (Auerbach, 1951). Moreover, the structural consequences of mutations, especially in complex biomolecular assemblies, are not always easy to interpret experimentally due to limitations in resolution and extensive obligations of time and resources and the availability of rapid structure screening techniques (McCafferty and Sergeev, 2017; Masso and Vaisman, 2008; Masso et al., 2006). These challenges significantly slow down the pace of mutation-driven discovery, emphasizing the growing need for complementary *in silico* tools that are capable of high-throughput mutational modeling and analysis (Masso and Vaisman, 2008; Masso et al., 2006). A thorough understanding of the effects of mutations requires not only detailed knowledge of primary sequences but also access to accurate structural data of biomolecular systems. However, introducing and analyzing multiple mutations across large and complex structures, particularly in a combinatorial manner, remains a significant challenge using conventional computational tools. To facilitate efficient *in silico* mutagenesis, we previously developed CHIMERA_NA, a command-line tool specifically designed for mutating nucleic acid structures (Pant, 2024a). This shell-based utility tool accepted standard structural format (PDB) and required basic user input, such as chain ID, residue ID, and the desired nucleotide information. While CHIMERA_NA was optimized for nucleic acids and served as a valuable tool for structure-based mutation modeling, its command-line interface, which required users to be comfortable with scripting environments and dependency on the now outdated UCSF Chimera posed usability challenges, particularly for non-computational users.

Existing platforms such as PyMOL (DeLano, 2002), VMD (Humphrey et al., 1996) and Chimera (Pettersen et al., 2004)/ChimeraX (Pettersen et al., 2021) provide basic structural visualization and limited mutagenesis functions, however, not well-suited for high-throughput applications. Moreover, these tools typically lack integrated workflows, batch-processing capabilities, and intuitive interfaces necessary for non-expert users to perform large-scale mutational analyses efficiently. Other existing computational mutagenesis tools, both academic and commercial, provide valuable capabilities for modeling mutations but remain largely protein-centric, workflow-fragmented, and limited in scalability and usability, particularly for nucleic acids and biomolecular complexes. Widely used academic platforms such as RosettaDDGPrediction (Sora et al., 2023), FoldX (Schymkowitz et al., 2005), MODELLER (Webb and Sali, 2016), and AlphaFold (Jumper et al., 2021) can be used for mutagenesis, however they primarily focus on protein mutations and either lack native support for nucleic acid mutagenesis or require extensive scripting, expert knowledge, and manual intervention to perform even basic modifications. Commercial suites including BioLuminate/Prime (Bhachoo and Beuming, 2017), MOE (Vilar et al., 2008), ICM-Pro (Abagyan et al., 1994), ProModel (Benson, 1997) and BIOVIA Discovery Studio (Baroroh et al., 2023) offer more user-friendly interfaces and improved support for protein–nucleic acid complexes; however, their nucleic acid mutation capabilities remain limited, largely restricted to single base substitutions at a time. In addition, these platforms are proprietary, expensive, and often require workflow customization or scripting to achieve batch processing, which limits accessibility and reproducibility. To overcome these limitations and expand the functionality beyond nucleic acids, we introduce BioMutation, a standalone, unified, open-source, interactive graphical user interface (GUI) designed to perform high-throughput systematic *in silico* mutagenesis of proteins, nucleic acids, and their complexes in an easy-to-execute UI environment without requiring scripting or advanced computational expertise. BioMutation enables users to easily introduce both user-defined mutations and systematic class-wise mutations in proteins or their complexes, as well as custom nucleotide substitutions in nucleic acids or di-/tri-/tetrameric mutations in DNA. The GUI automatically outputs a library of mutant structures in standard formats (PDB, MOL2, mmCIF), making them ready for downstream computational studies such as molecular docking and molecular dynamics simulations. For analyzing the changes in physical and chemical features of mutated biomolecules, the GUI includes a Google Colab (Bisong, 2019) notebook accessible via the Structure Analyzer tab in BioMutation that helps the user analyze and compare changes in different features of the biomolecules after the introduction of mutations.

## Methods

**FIGURE 1:**
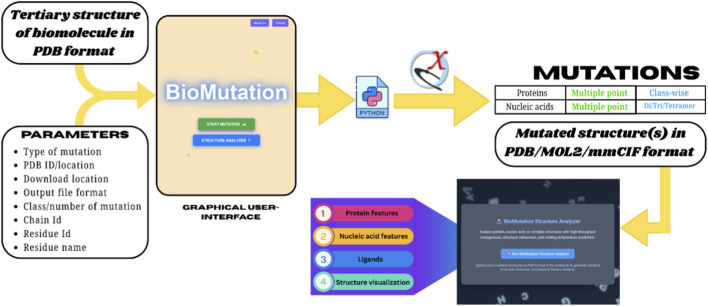
The outline of the BioMutation GUI demonstrates the overall working protocol of the tool, including the input queries, expected output files and further workflow for analysis of mutated structures.

BioMutation is a user-friendly graphical interface designed to streamline the process of biomolecular mutagenesis through an intuitive, step-by-step workflow. It iteratively utilizes the mutagenesis engines of UCSF ChimeraX, namely, swapna for nucleic acid point mutations and swapaa for amino acid point mutations, based on user-defined requirements. Built using modern web technologies (HTML, CSS, JavaScript), BioMutation is lightweight, runs locally on standard systems, and requires no installation beyond ChimeraX. With its user-friendly design and expanded capabilities, BioMutation can be a valuable GUI-based platform for high-throughput mutagenesis across biomolecular systems. The architecture and operational workflow of BioMutation are illustrated in Figure 1.

### Workflow summary

The BioMutation workflow begins with the input of a biomolecular tertiary structure in PDB format, either uploaded locally or retrieved from the RCSB PDB webserver using the PDB code. Through an intuitive graphical user interface, users define mutation parameters, such as biomolecule type, mutation scheme (single, multiple, class-wise, or di-/tri-/tetrameric substitutions), chain ID, residue or nucleotide position, and desired output format. Based on these inputs, BioMutation automatically generates a ChimeraX-compatible Python script that executes substitution-based mutations and produces a library of mutated structures in PDB, MOL2, or mmCIF formats. The resulting mutant structures can then be directly analyzed using the integrated BioMutation Structure Analyzer module, which extracts physicochemical features, visualizes structures in three dimensions, and facilitates comparative assessment of mutation-induced changes, thereby enabling a streamlined end-to-end pipeline for high-throughput *in silico* mutagenesis and analysis.

### Installation

The installation of the BioMutation tool on the local system requires users to download and unzip the BioMutation_codes.zip file from the GitHub repository https://github.com/Computational-biolab/BioMutation, and simply run the start.html file to start the tool. All the HTML files are linked and direct users to later steps. A tutorial is also added on the home page that users may access to understand the overall execution of the tool.

### Performing mutation(s)

Users begin by selecting the biomolecule type (nucleic acid or protein), followed by the desired mutation scheme. Supported mutation types include single or multiple point mutations, as well as class- or combination-based mutagenesis, such as di-, tri-, or tetranucleotide substitutions in DNA, or residue class-specific substitutions (e.g., hydrophobic, polar, aromatic, acidic, and basic) in proteins (Fisher, 2001). The tool accepts either a PDB ID or a local PDB file as input and allows users to specify mutation parameters such as chain ID, residue ID, and the replacement base or amino acid. The output can be generated in PDB, MOL2, or mmCIF formats, as desired by the user. A dynamic summary table displays user input in real time and can be modified before execution. Upon confirmation, BioMutation generates a customized Python script compatible with UCSF ChimeraX, which automates the mutation process and saves the resulting mutant structure(s) to the specified local directory. The mutant structure(s) can be utilized for further computational studies. All the types of supported mutations compatible with different biomolecules have been summarized in Table 1.

**TABLE 1:** Summary of all the supported mutations induced by BioMutation on different biomolecules.

Biomolecular system	Mutation type	Mutation description	Class-wise/Combinatorial support	Strand or chain handling	Output formats
Protein	Single-site substitution	Replacement of a single amino acid at a user-defined position	No	Protein backbone preserved	PDB, MOL2, mmCIF
Protein	Multi-site substitution	Substitution of multiple user-specified amino acids within a chain	No	Protein backbone preserved	PDB, MOL2, mmCIF
Protein	Class-wise substitution	Systematic substitution using predefined amino acid classes	Yes	Protein backbone preserved	PDB, MOL2, mmCIF
DNA	Single-site substitution	Replacement of a single nucleotide at a specified position	No	Complementary base (optionally specified)	PDB, MOL2, mmCIF
DNA	Multi-site substitution	Replacement of multiple nucleotides at specified positions	No	Complementary bases (user-defined)	PDB, MOL2, mmCIF
DNA	Combinatorial substitution	Di-, tri-, or tetranucleotide substitutions across consecutive positions	Yes	Complementary strand-pairing is preserved	PDB, MOL2, mmCIF
RNA	Single-site substitution	Replacement of a single nucleotide at a specified position	No	RNA backbone preserved	PDB, MOL2, mmCIF
RNA	Multi-site substitution	Replacement of multiple nucleotides within an RNA chain	No	RNA backbone preserved	PDB, MOL2, mmCIF
Protein and nucleic acid complex	Combined substitution	Substitutions in a single biomolecule at a time	Yes	Either nucleic acid or protein handled at a time. All other molecules remain conserved, if present	PDB, MOL2, mmCIF

The table summarizes the types of biomolecule, types of mutations, description, class-wise/combinatorial support, strand or chain handling and output format.

### Feature analysis

**FIGURE 2:**
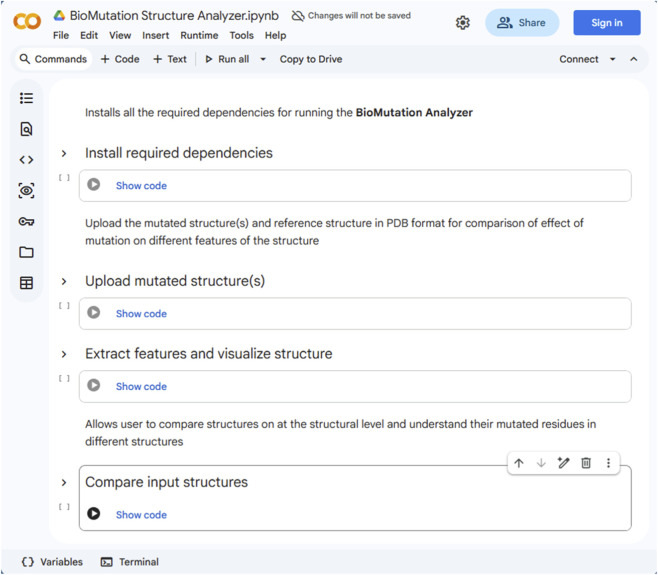
Homepage of BioMutation structure analyzer Google colab for biomolecules feature analysis. The colab notebook is designed for users to directly install required libraries, input structures for feature extraction and compare structures quantitatively and visually.

In silico assessment of mutations requires not only generating altered structures but also quantitative evaluation of how residue or base replacements perturb the physicochemical landscape of the biomolecule. In the BioMutation Structure Analyzer module, a defined set of physicochemical features, including sequence, length, molecular weight, isoelectric point, aromaticity, instability index, average flexibility, hydropathicity (GRAVY), secondary structure fractions, extinction coefficients, and an approximate melting temperature, are systematically predicted to quantify the structural consequences of substitution-based mutations and compare wild-type and mutant biomolecules. This Google Colab-based module is used for comparing structures with induced mutations via BioMutation with their original structures and may be used as a standalone module for feature extraction of multiple input PDBs. The module supports feature extraction from proteins and nucleic acids present in the input structure. For feature extraction using BioMutation Structurer Analyzer, a Google Colab (Bisong, 2019) notebook is developed that implements an integrated pipeline for automated structural feature extraction, quantitative biomolecular characterization, and interactive visualization of macromolecular structures derived from Protein Data Bank (PDB) files within a Jupyter-based environment. Initially, it imports established bioinformatics and visualization libraries (BioPython (Cock et al., 2009), pandas (McKinney, 2011), py3Dmol, and ipywidgets) to ensure robust parsing, analysis, and user interaction. For each uploaded PDB file, the structure is parsed using BioPython (Cock et al., 2009) PDBParser (Hamelryck and Manderick, 2003), and the first model is analyzed to identify and classify chains into proteins, nucleic acids (DNA or RNA), and ligands based on residue names and heteroatom flags. Protein chains are reconstructed into continuous peptide sequences using the PPBuilder module of Bio.PDB for protein sequence extraction, followed by comprehensive physicochemical analysis via the ProteinAnalysis module, yielding metrics such as isoelectric point (Sillero and Ribeiro, 1989), aromaticity, instability index, flexibility, hydropathicity (GRAVY) (Kyte and Doolittle, 1982), secondary structure fractions, extinction coefficients (Gill and Von Hippel, 1989), and an approximate melting temperature. Nucleic acid chains are similarly processed to extract sequences, length, GC content, molecular weight, and estimate melting temperature using the Wallace rule (García-Ballesteros et al., 2023). These features have an important biological significance in understanding different physical and chemical properties of the biomolecule. The isoelectric point (pI) captures alterations in net charge introduced by substituting acidic, basic, or neutral residues, which can significantly influence electrostatic interactions, solubility, and binding behaviour. Aromaticity quantifies changes in aromatic residue content, advising modifications to hydrophobic packing and π–π interactions that often stabilize protein cores or nucleic acid binding interfaces. The instability index provides an empirical estimate of the impact of substitutions on protein stability, highlighting induced mutations that may influence the structure to unfolding or degradation. Flexibility metrics reflect changes in local and global conformational mobility induced by residue replacement, which may affect functional dynamics and allosteric regulation. Hydropathicity (GRAVY) measures shifts in overall hydrophobic or hydrophilic character resulting from substitutions, with implications for folding, solvent exposure, and intermolecular interactions. Secondary structure fractions (α-helix, β-sheet, and coil) enable evaluation of whether substitutions bias the structural ensemble toward more ordered or disordered conformations. Extinction coefficients estimate changes in UV absorbance arising from altered aromatic residue composition or environment, facilitating experimental cross-validation. Finally, an approximate melting temperature serves as an indicator of relative thermal stability, allowing comparison of stabilizing versus destabilizing substitutions. In case of nucleic acids, the estimated melting temperature is calculated using the Wallace rule (García-Ballesteros et al., 2023), a simple empirical relationship that correlates base composition with duplex stability [T_m_ (°C) = 2 × (A + T) + 4 × (G + C)]. All extracted features are organized into structured pandas (McKinney, 2011) DataFrames for proteins, nucleic acids, and ligands, which are then exported as CSV files. Concurrently, the three-dimensional structure is visualized using py3Dmol, rendering macromolecules in a cartoon representation with spectrum colouring, while detected ligands are highlighted as magenta spheres to emphasize functional components. Finally, the code assembles an interactive graphical layout using ipywidgets, displaying scrollable feature tables alongside the 3D molecular viewer. The BioMutation Structure Analyzer Google Colab is shown in Figure 2. Together, these complementary features enable a systematic and interpretable evaluation of mutation-driven structural perturbations, supporting high-throughput comparative analysis within the BioMutation Structure Analyzer workflow. This approach is well suited for comparative analysis of closely related mutant sequences, enabling efficient screening of stabilizing or destabilizing base/amino acid substitutions in nucleic acid/protein systems.

## Results and discussion

Designed for both novice and experienced computational users, BioMutation enables seamless input of mutation parameters through a guided interface and supports both direct command generation and the creation of editable Python scripts for execution within the ChimeraX environment. This dual functionality enables both interactive and scripted workflows, accommodating various user preferences and requirements. The following cases illustrate diverse applications of BioMutation across multiple mutation strategies.

### Case 1

**FIGURE 3:**
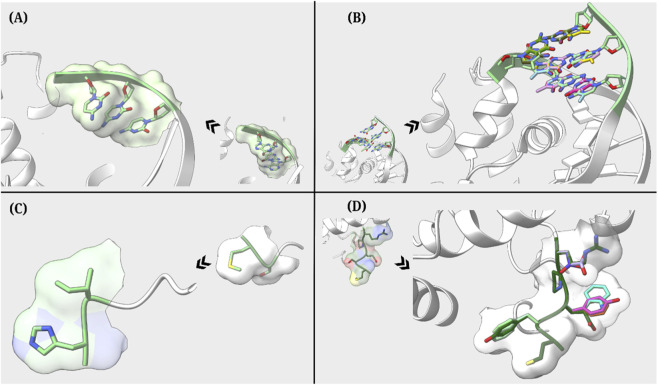
BioMutation GUI-generated mutant structures, along with their reference structures (towards the centre), are shown (**(A)** Case 1, **(B)** Case 2, **(C)** Case 3, **(D)** Case 4). The mutated regions are presented in stick representation.

To assess site-specific mutations within a DNA chain bound to the Cryo-EM structure of the *Escherichia coli* replicative DNA polymerase complex (Fernandez-Leiro et al., 2015) (PDB ID: 5FKV), the BioMutation GUI was employed. For introducing mutations into chain P at residue positions 1, 2, and 3, substituting each residue with a cytosine (C). The BioMutation tool generated a mutated structure with cytosine base at the three desired locations, reflecting cytosine incorporation at the targeted sites while preserving the native spatial geometry and atomic connectivity of the original structure by selecting the optimal rotamer based on Dunbrack rotamer library (Dunbrack and Karplus, 1993) for a given position based on criteria such as minimum atomic clashes, highest probability, maximum hydrogen bonds, or best fit to density maps. The resulting model was saved in .mmCIF format, ensuring compatibility with downstream applications such as structure refinement and molecular visualization. Comparative structural representations of the original and mutated complexes are presented in Figure 3. This mutation workflow demonstrates the utility of BioMutation in nucleic acid engineering, providing a potential framework for designing and studying the structural and functional consequences of specific base substitutions. Such targeted modifications have broad applications in understanding DNA-protein interactions, guiding mutagenesis experiments, and designing synthetic biological systems.

### Case 2

Precise terminal mutations are important for investigating sequence-dependent effects on DNA structure and interactions. To understand this, a crystal structure of DnaA domainIV complexed with DnaAbox DNA (Fujikawa et al., 2003) was selected (PDB ID 1J1V) and residue IDs 113, 112 and 111 of chain B and complementary chain C residues at residue IDs 201, 202 and 203 were targeted. Using the BioMutation GUI, all possible triple base pair combinations, including complementary pairs, were generated. BioMutation successfully produced 64 distinct PDB files, each representing a unique set of mutations. This case presents the efficiency of BioMutation in generating DNA variants for structural and functional studies with user-defined mutations at desired residue sites. The overlay of the structures generated is shown in Figure 3 along with the reference structure.

### Case 3

To explore multiple site-specific amino acid substitutions within a protein chain, the Cryo-EM structure of the *E. coli* DNA polymerase bound to DNA (Fernandez-Leiro et al., 2015) (PDB ID: 5KVF) was selected. Protein chain A was targeted for mutation, where residue 1 was substituted with histidine (His) and residue 2 was substituted with isoleucine (Ile). The BioMutation tool was used to generate a Python script, which was subsequently executed within the ChimeraX environment to apply the specified mutations. The resulting structure reflected both amino acid substitutions while preserving side chain geometry and atomic connectivity. The mutated model was saved in PDB format for further analysis. Comparative visualization of the original and mutated structures is presented in Figure 3, highlighting the efficiency of BioMutation GUI to introduce multiple mutations at desired residues in the structures while maintaining the structural integrity.

### Case 4

To perform class-wise mutations based on biochemical properties, the Cryo-EM structure of the *E. coli* DNA polymerase bound to DNA (Fernandez-Leiro et al., 2015) (PDB ID: 5KVF) was selected. Chain A of the protein was targeted, with residue IDs 2 and 3 mutated individually to all members of the aromatic class (phenylalanine, tyrosine, and tryptophan). Additionally, residue ID 5 was mutated to acidic amino acids (aspartic acid and glutamic acid). BioMutation successfully generated all permutations of the desired class-wise mutations, resulting in a total of eight unique PDB structures. Each PDB file represented a distinct mutation scenario, greatly facilitating the creation of a mutant library suitable for *in silico* screening and docking studies. The overlay of the original and mutated structures is shown in Figure 3, highlighting the effectiveness of BioMutation in systematically generating class-based mutation variants.

The presented test cases collectively demonstrate the versatility and flexibility of the BioMutation tool in introducing substitution-based *in silico* mutations at user-defined single or multiple residue positions in both nucleic acid and protein structures. The mutated biomolecular structures by BioMutation GUI and can be integrated into a range of downstream computational pipelines. Mutated structures can be used to investigate the dynamic behaviour, structural stability, and conformational flexibility of biomolecules under varying physiological conditions. This enables the assessment of mutation-induced perturbations in structure and function. These structures may serve as valuable input datasets for training ML models aimed at predicting mutational effects on protein stability, nucleic acid base-pairing fidelity, binding affinities, or enzymatic activities. However, BioMutation produces initial, unrefined *in silico* structures and does not perform post-mutation energy minimization, structural refinement, or stability assessment. Users are therefore advised to apply external minimization or molecular dynamics protocols to evaluate structural stability for downstream validation. Also, the tool supports only substitution mutations and does not allow insertions or deletions.

### Validation

To validate the accuracy of BioMutation across different biomolecular systems, four case studies were conducted. Four PDB structures were selected from RCSB PDB, and a sequentially similar structure was derived using the BLAST algorithm for each case. Using the BioMutation GUI, the similar structures were mutated at different residue locations to match the sequence with the initially selected PDB structures. The structures were overlaid, and all-atom RMSD values (local and global) were determined to show the similarity among the existing structure residues and mutated PDB residues.

**FIGURE 4:**
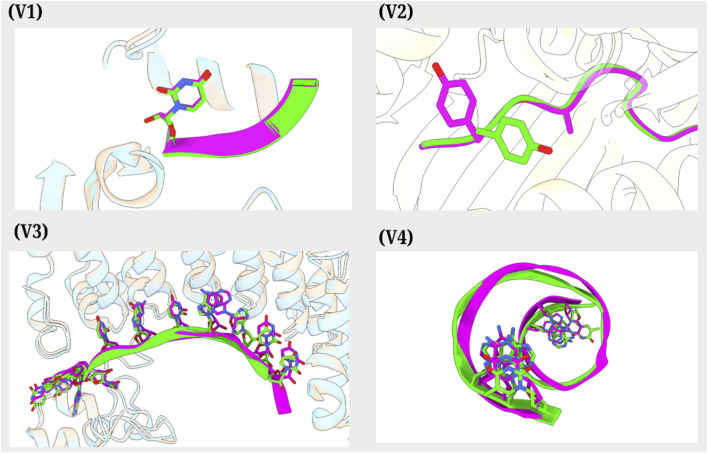
Overlay of experimentally derived structures (green) from RCSB PDB and mutated structures (violet) generated via BioMutation ((V1) Case 1, (V2) Case 2, (V3) Case 3, (V4) Case 4).

In the first validation case (V1), a single nucleotide at the 12th residue A of the 12-mer siRNA of a protein-RNA complex (PDB ID: 4NGC) was mutated to U and overlapped with an existing similar structure from RCSB PDB (PDB ID: 4NGB). Overlay of structures showed significant overlap, including at the mutation site (global RMSD: 0.18 Å and local RMSD: 0.04 Å). In Figure 4, the mutated residue (stick representation) and the overall structure (cartoon) are depicted in violet, while the existing similar structure is shown in green. This suggests the application of BioMutation in introducing site-specific mutations while maintaining structural integrity, which is essential for functional and structural RNA studies. In the second validation case (V2), a peptide structure within a protein-peptide complex was mutated at two locations, 6 and 8, to ALA and TYR, respectively, to match the already known peptide sequence. Alignment highlighted that the mutated residues (violet; stick form; PDB ID: 1QSF) matched the residues of the reference structure (green; PDB ID: 1QRN) with global RMSD: 0.43 Å and local RMSD: 0.33 Å, highlighting the suitability of BioMutation for peptide engineering, epitope mapping, and therapeutic design. In the third validation case (V3), the entire RNA chain was mutated to match a reference structure. The mutated chain (violet) and its residues (stick form) (PDB ID: 5KL1) aligned closely with the reference structure (green; PDB ID: 5KL8) with global RMSD: 0.74 Å and local RMSD: 0.71 Å, suggesting the ability of BioMutation to perform extensive RNA and other nucleic acid modifications, which is critical for applications in RNA engineering and nucleic acid-based synthetic biology. In the fourth validation case (V4), multiple mutations were introduced at the terminal ends of two DNA strands to align with a known reference sequence. Structural comparison showed that the mutated residues (violet, stick form) (PDB ID: 8T4U) overlapped with the reference residues (green; PDB ID: 7L4C). The RMSD calculations for V4 were not performed due to the pending release of the 8T4U coordinates on RCSB webserver. These validation cases collectively establish BioMutation as a reliable tool for applying desired single-site and multi-site mutations across RNA, DNA, and peptide systems by substituting side chains of desired residues by selecting the optimal rotamer based on Dunbrack rotamer library (Dunbrack and Karplus, 1993) for a given position based on criteria such as minimum atomic clashes, highest probability, maximum hydrogen bonds, or best fit to density maps, enabling wide applications in molecular biology, bioengineering, and drug discovery.

### Structure analyzer

The BioMutation Structure Analyzer module gives users a platform to understand different physicochemical changes in the overall structures after inducing desired mutations. A Google Colab (Bisong, 2019) notebook is developed that implements an integrated pipeline for automated structural feature extraction, quantitative biomolecular classification, and interactive visualization of macromolecular structures derived from Protein Data Bank (PDB) files within a Jupyter-based environment. For the validation of the module, modified PDB structures and their original reference structures from test cases were compared based on different parameters implemented in the Structure Analyzer module.

We utilized validation case 3 for demonstrating the change in features after performing the desired mutations. The RNA component of the Pumilio-Nos-hunchback RNA complex (PDB ID: 5KL1) was mutated to a reference sequence (PDB ID: 5KL8) by substituting 4 residues of the RNA structure. The reference and the mutated RNA structures were compared using the Structure Analyzer module and the results are reported in Table 2.

**TABLE 2:** The physicochemical features of the RNA chain from the Pumilio-Nos-hunchback RNA complex (PDB ID: 5KL1) before and after mutations are reported.

Structure ID	Chain ID	NA type	Sequence	Length	GC Content (%)	Mol. Weight (Da)	Estimated melting temp (°C) (Wallace rule)
5KL1	C	RNA	**A**A**A**UUGUA**CA**UA	12	16.67	3868.3	28
5KL1_mutated	C	RNA	**U**A**U**UUGUA**AU**UA	12	8.33	3823.21	26

Comparative analysis of the RNA segment of the original structure (Structure ID: 5KL1) and its mutated counterpart (5KL1_mutated) reveals distinct physicochemical changes arising due to substitution-based mutations, with sequence length remaining constant at 12 nucleotides. The reduction in GC content from 16.67% to 8.33% indicates replacement of guanine or cytosine residues with adenine or uracil, leading to a measurable decrease in molecular weight from 3868.3 Da to 3823.21 Da. This compositional shift is accompanied by a decrease in the estimated melting temperature from 28 °C to 26 °C, as calculated using the Wallace rule, reflecting reduced duplex stability due to weaker AU base pairing relative to GC interactions. Thus, the observed physicochemical alterations highlight how targeted base substitutions can alter RNA stability, emphasizing the relevance of systematic mutational analysis for understanding sequence–structure–function relationships in RNA-mediated biological processes.

## Conclusion

BioMutation is an easy-to-use GUI, designed to induce mutations in proteins, peptides, nucleic acids and their complexes. It provides a user-friendly platform for computational mutagenesis by offering a portable, automated, and user-centric graphical user interface for performing targeted and combinatorial mutations across a wide array of biomolecules, including DNA, RNA, proteins, and their complexes. Built upon the iterative use of mutagenesis engines of UCSF ChimeraX, namely, swapna for nucleic acid point mutations and swapaa for amino acid point mutations, BioMutation simplifies the mutation workflow through an interactive, self-contained HTML-based platform that requires no additional installation or configuration. The GUI enables users to generate site-specific or class-wise mutations by leveraging UCSF ChimeraX mutation capabilities through curated Python scripts. It accommodates structural input either via PDB ID or local file location and supports export in widely used structural formats (PDB, MOL2, mmCIF), allowing direct downstream application in state-of-the-art computational techniques such as molecular dynamic simulations (Pant et al., 2020; Pant, 2024b; Pant et al., 2017; Pant et al., 2023; Hollingsworth and Dror, 2018; Pant, 2024c; Sharma et al., 2023; Pant and Leese, 2023; Pant, 2022), *in silico* aptamer optimization techniques (Gupta et al., 2025; Buglak et al., 2020; Yu et al., 2026; Zheng et al., 2026), docking studies (Liu et al., 2025), machine learning pipelines (Rimal et al., 2025; Kopac, 2025; Seth and Bhattacharya, 2025), and structural-functional analyses (Parvizpour et al., 2020). The tool also offers users an opportunity to compare and analyze changes in physical and chemical features using the Structure Analyzer module of BioMutation, using Python libraries and functions to extract key features and compare the effect of mutations on the overall biomolecule. It is important to note that BioMutation focuses exclusively on structural mutation generation (i.e., providing initial coordinates) and feature comparison, and does not perform energy minimization or stability scoring; such analyses, if required, are intended to be carried out using appropriate downstream computational tools. BioMutation has been validated through multiple practical case studies, illustrating its flexibility and efficiency in modeling diverse mutational scenarios. Its intuitive design bridges the gap between computational complexity and biological utility, serving both novice users and experienced researchers. By enabling high-throughput, reproducible mutagenesis with minimal manual intervention, BioMutation provides a powerful platform for structural biology, protein engineering, and synthetic biology research. As an open-access resource, BioMutation is distributed in a portable format and is accompanied by a comprehensive user tutorial to facilitate widespread adoption. The GUI holds promise for accelerating hypothesis-driven experimentation, guiding rational biomolecular design, and advancing understanding of mutation-induced structural dynamics.

## Data Availability

The datasets presented in this study can be found in online repositories. The names of the repository/repositories and accession number(s) can be found below: https://github.com/Computational-biolab/BioMutation.
